# Stroke prevention within primary care: management of atrial fibrillation using oral anticoagulation

**DOI:** 10.1590/1516-3180.2018.1364110718

**Published:** 2018-07-21

**Authors:** Paulo Andrade Lotufo

**Affiliations:** I MD, DrPH. Full Professor, Department of Internal Medicine, Faculdade de Medicina da Universidade de São Paulo (FMUSP), São Paulo (SP), Brazil.

Chronic atrial fibrillation is a common heart arrhythmia closely associated with aging.^1^ In Brazil, in the city of São Paulo, a door-to-door survey conducted in 2006 in the catchment area of a primary care unit showed that the prevalence of atrial fibrillation was 2.7% among people aged 65 years and older.^2^^,^^3^ Another study in the state of Minas Gerais with data from primary care units showed similar prevalence,^4^ and these surveys revealed that the frequency of atrial fibrillation increased exponentially according to age strata, such that, for example, for people aged 80 to 89, the prevalence ranged from 7.5% to 9.8%.^2^^,^^3^^,^^4^ Despite this epidemiological profile, atrial fibrillation is still not included as a public health issue, due to the paucity of epidemiological studies addressing elderly individuals’ health issues.^1^

On the other hand, accurate clinical knowledge of atrial fibrillation (formerly known as auricular fibrillation) has been available since the 1930s. In the seminal textbook *Heart Disease*, by Paul Dudley-White (1886-1973), published in 1937, the author stated “that the establishment of auricular fibrillation is often obscure in its origin. In most cases, there is a significant degree or type of heart disease or an important toxic agent or disease of some other nature, but sometimes there is no such cause and these individuals seem to be perfectly healthy without heart disease”. Dudley-White, who is regarded as the father of American cardiology, explained that palpitation was “the characteristic symptom of atrial fibrillation”, and he stated that “the observer, whether nurse or doctor, must be taught in such cases to record not only the radial pulse rate but, what is much more important, the apex heart rate also”. Interestingly, Dudley-White recognized that “electrocardiogram is of the greatest assistance in confirming the diagnosis of auricular fibrillation, although this confirmation is often unnecessary”.^5^

Over the next five decades, atrial fibrillation was only correlated with aging and was treated through relieving the symptoms. However, in the 1990s, the first results from the Framingham Heart Study threw new light on this condition.^6^ Firstly, the scientists analyzing this cohort were able to identify from among the risk factors for atrial fibrillation that age was the most significant risk factor for atrial fibrillation, in comparisons with body mass index, intake of alcoholic beverages, smoking, diabetes and hypertension.^7^ In the most recent analysis on the cohort (1998-2007), in comparison with individuals aged 50 to 59 years, the chance of having atrial fibrillation was five times greater for participants aged 60 to 69, seven times greater for those aged 70 to 79 and nine times greater for those aged 80 to 89 years.^6^ Secondly, the outcomes of atrial fibrillation were very severe because this increased the incidence of stroke, heart failure and dementia. Cerebrovascular events consequent to atrial fibrillation are more often fatal or disabling, and present greater risk of dementia than do stroke events with etiologies that do not include atrial fibrillation.^8^^,^^9^

Randomized controlled trials comparing warfarin to placebo or control that were conducted two decades ago clearly showed that warfarin reduced ischemic stroke by 64% and all-cause deaths by one quarter.^10^ Inevitably, use of warfarin and other vitamin K antagonists (VKA) became the standard of care for stroke prevention. However, use of warfarin is ambivalent, in that it requires precise anticoagulation monitoring of INR (international normalized ratio) prothrombin time, to keep this within a relatively narrow therapeutic range and avoid the risk of severe bleeding.^11^ Hence, the emphasis in prevention of stroke due to atrial fibrillation is on identifying high-risk atrial fibrillation patients for whom the benefit of anticoagulation surpasses the risk of life-threatening bleeding events. More recently, the introduction of non-VKA oral anticoagulants (NOACs), including direct thrombin inhibitors and factor Xa inhibitors, without the need to control for prothrombin time should have led to changes to the perspectives for the approaches towards atrial fibrillation and stroke.^11^ However, most public healthcare systems cannot afford to supply NOACs for long-term use.

Consequently, in the Brazilian National Health System, there is a need to introduce a pragmatic program to deal with atrial fibrillation in association with anticoagulation that does not necessarily involve screening individually for atrial fibrillation but enables greater depth of evaluation on people who seek care complaining of palpitations. To reach this objective, one of the tasks should be to teach healthcare providers to examine the radial and apex heart rate in people who have been identified as presenting high risk of stroke, such as in the way proposed by Paul Dudley-White.^5^ Moreover, all programs relating to hypertension and diabetes control need to identify people with atrial fibrillation by means of pulse palpation accompanied by a 12-lead resting electrocardiogram.^11^

To achieve this, all healthcare providers within the primary care setting will need to introduce the following mnemonic procedures:

1. Estimation of stroke risk among people with atrial fibrillation, using tools such as CHA2DS2-VASc, i.e. “Congestive heart failure, Hypertension, Age ≥ 75, Age between 65 and 74, Diabetes mellitus, prior Stroke, Transient Ischemic Attack [TIA] or Thromboembolism, Vascular disease, Sex female”.^13^
2. Estimation of bleeding risk by applying HAS-BLED scores, i.e. “Hypertension, Abnormal renal or liver function, Stroke, Bleeding, Labile INR, Elderly (age > 65 years) and Drugs (alcohol)”.^13^
3. Prediction of poor control through warfarin therapy by applying the SAMe-TT2R2 tool, i.e. “Sex (female), Age (<60 years), Me (medical history), Treatment (interacting drugs, e.g. amiodarone for rhythm control), Tobacco use (within two years), Race (non-European ancestry)”.^14^

Several algorithms that enable safe adoption of anticoagulation within primary care are available. However, the approach towards people with atrial fibrillation implies reduction of the symptoms through prescribing beta-blockers and decreasing the burden of other risk factors like hypertension and dyslipidemia.^11^

A more detailed nationwide registry of atrial fibrillation is underway at 80 sites in Brazil, with follow-up on 5,000 patients with this arrhythmia. This will help to clarify some aspects of its prevention at primary care level.^15^

However, the challenge pointed out previously in this Journal is to put cardiovascular prevention into primary care.^16^ This is an action that will effectively reduce the social gap regarding mortality,^17^ morbidity^18^ and disability^19^ due to stroke, which is still a neglected disease in Brazil.^20^
